# Levels of extracellular matrix metabolites are associated with changes in Ankylosing Spondylitis Disease Activity Score and MRI inflammation scores in patients with axial spondyloarthritis during TNF inhibitor therapy

**DOI:** 10.1186/s13075-022-02967-8

**Published:** 2022-12-23

**Authors:** Signe Holm Nielsen, Shu Sun, Anne C. Bay-Jensen, Morten Karsdal, Inge Juul Sørensen, Ulrich Weber, Anne Gitte Loft, Gina Kollerup, Gorm Thamsborg, Ole Rintek Madsen, Jakob Møller, Mikkel Østergaard, Susanne Juhl Pedersen

**Affiliations:** 1 Nordic Bioscience, Herlev, Denmark; 2 Biotechnology and Biomedicine, Technical University of Denmark, Lyngby, Denmark; 3 Copenhagen Center for Arthritis Research, Center for Rheumatology and Spine Diseases, Rigshospitalet, Glostrup, Denmark; 4 Department of Clinical Medicine, University of Copenhagen, Copenhagen, Denmark; 5 Danish Hospital for Rheumatic Diseases, University Hospital of Southern Denmark, Sønderborg, Denmark; 6 Practice Buchsbaum, Rheumatology, Schaffhausen, Switzerland; 7 Departments of Rheumatology, Hospital Lillebælt, Vejle, Denmark; 8 Aarhus University Hospital, Aarhus, Denmark; 9 Department of Radiology, Herlev Hospital, Copenhagen, Denmark

## Abstract

**Background/purpose:**

In axial spondyloarthritis (axSpA) inflammation of the sacroiliac joints and spine is associated with local extracellular matrix (ECM) remodeling of affected tissues. We aimed to investigate the association of ECM metabolites with treatment response in axSpA patients treated with TNF-α inhibitory therapy for 46 weeks.

**Methods:**

In a prospective clinical study of axSpA patients (*n*=55) initiating a TNF inhibitor (infliximab, etanercept, or adalimumab), serum concentrations of formation of type I (PRO-C1), type III (PRO-C3), and type VI (PRO-C6) collagen; turnover of type IV collagen (PRO-C4), and matrix-metalloproteinase (MMP)-degraded type III (C3M) collagen, MMP-degraded type IV (C4M), type VI (C6M), and type VII (C7M) collagen, and cathepsin-degraded type X collagen (C10C), MMP-mediated metabolite of C-reactive protein (CRPM), citrullinated vimentin (VICM), and neutrophil elastase-degraded elastin (*EL*-*NE*) were measured at baseline, week 2, week 22, and week 46.

**Results:**

Patients were mostly males (82%), HLA-B27 positive (84%), with a median age of 40 years (IQR: 32–48), disease duration of 5.5 years (IQR: 2–10), and a baseline Ankylosing Spondylitis Disease Activity Score (ASDAS) of 3.9 (IQR: 3.0–4.5).

Compared to baseline, PRO-C1 levels were significantly increased after two weeks of treatment, C6M levels were significantly decreased after two and 22 weeks (repeated measures ANOVA, *p*=0.0014 and *p*=0.0015, respectively), EL-NE levels were significantly decreased after 2 weeks (*p*=0.0008), VICM levels were significantly decreased after two and 22 weeks (*p*=0.0163 and *p*=0.0374, respectively), and CRP were significantly decreased after two and 22 weeks (both *p*=0.0001). Baseline levels of PRO-C1, PRO-C3, C6M, VICM, and CRP were all associated with ASDAS clinically important and major improvement after 22 weeks (ΔASDAS ≥1.1) (Mann–Whitney test, *p*=0.006, *p*=0.008, *p*<0.001, <0.001, <0.001, respectively), while C6M, VICM and CRP levels were associated with ASDAS clinically important and major improvement after 46 weeks (ΔASDAS ≥2.0) (*p*=0.002, *p*=0.044, and *p*<0.001, respectively). PRO-C1 and C6M levels were associated with a Bath AS Disease Activity Score (BASDAI) response to TNF-inhibitory therapy after 22 weeks (Mann–Whitney test, *p*=0.020 and *p*=0.049, respectively). Baseline levels of PRO-C4 and C6M were correlated with the total SPARCC MRI Spine and Sacroiliac Joint Inflammation score (Spearman’s Rho *ρ*=0.279, *p*=0.043 and *ρ*=0.496, *p*=0.0002, respectively).

**Conclusions:**

Extracellular matrix metabolites were associated with ASDAS response, MRI inflammation, and clinical treatment response during TNF-inhibitory treatment in patients with axSpA.

## Background

Axial spondyloarthritis (axSpA) is a chronic inflammatory disease that is characterized by inflammation in the sacroiliac joints and spine, and over time some patients progress from non-radiographic axSpA to radiographic axSpA (ankylosing spondylitis (AS)) [1, 2]. There is an unmet need to identify biomarkers that reflect disease activity in patients with axSpA [3]. To monitor disease activity in axSpA patients, the AS Disease Activity Score (ASDAS) was developed and is now widely used [4]. It combines a questionnaire with items regarding back pain, peripheral pain, and duration of morning stiffness with blood C-reactive protein (CRP) levels. However, there are some drawbacks of ASDAS, as it mainly reflects patients’ perspectives, and CRP is highly weighted in the score [5]. Magnetic resonance imaging (MRI) has emerged as a reliable imaging modality in axSpA to detect bone marrow edema (BME) and structural lesions. However, whether patients are scanned by MRI is highly dependent on the availability of MRI devices [6], and repeated MRIs in routine care are often not feasible. There is a lack of commonly available and objective measures to monitor changes in disease activity in patients diagnosed with axSpA. We hypothesized that biomarkers of extracellular matrix (ECM) remodeling might assist in the evaluation of disease activity and response to treatment in axSpA.

During the disease progression of axSpA, inflamed tissue is infiltrated by immune cells, which creates a disturbance in tissue homeostasis, where ECM proteins are formed but also degraded by the overexpression and activation of proteases, such as metalloproteinases (MMPs) and cathepsins [7]. Both the formation and degradation of the ECM are reflected in changes in circulating biochemical markers of ECM fragments, so-called neo-epitopes [8]. The 28 types of collagen are the major proteins in the body and are expressed in various tissues, including joint tissues [9]. In particular, type I, III, IV, VI, VII, and X collagen are expressed in joint tissues, including tendons, bone, and connective tissue. Type I collagen is the most abundant protein, as it is the major structural protein in bone. PRO-C1, measuring type I collagen formation (N-terminal pro-peptide of type I procollagen [PINP]), has been shown to be a biomarker of bone formation [10]. Type I and III collagen are the major ECM proteins in soft tissue, while type IV collagen is the major constituent of the basement membrane located below the epithelial/endothelial cells. The biomarker PRO-C3, which measures type III collagen formation and is a marker of fibrogenesis, has previously been shown to be associated with progressive systemic sclerosis [11]. Turnover of type IV collagen, quantified by PRO-C4, and the two MMP-mediated fragments of type III and IV collagen, C3M and C4M, respectively, are associated with response to TNF-a inhibitor therapy in inflammatory bowel disease [12]. Type VI collagen formation, quantified by PRO-C6, is elevated in patients with rheumatoid arthritis and predicts response to an anti-IL-6 receptor antibody in combination with methotrexate (MTX) after 16 weeks [13]. The MMP-mediated degradation fragment of type VI collagen, C6M, has previously been shown to be upregulated in AS [14]. Type VII collagen is a minor collagen anchoring the fibrils that connect the basement membrane to the underlying interstitial matrix. Degradation of type VII collagen can be quantified by the MMP-mediated fragment of type VII collagen, C7M, which has been found to be upregulated in systemic sclerosis [15]. Type X collagen is primarily expressed by hypertrophic chondrocytes [16], and the biomarker C10C, reflecting cathepsin-degraded type X collagen, is elevated in patients with osteoarthritis [17]. Neutrophil elastase-degraded elastin (EL-NE) is a potential biomarker for differentiating inflammatory bowel disease from irritable bowel syndrome [18]. It has been suggested that the MMP-mediated metabolite of CRP (CRPM) reflects local tissue inflammation [19, 20], and it has been associated with changes in disease activity in patients with AS [19]. Finally, vimentin (VICM), an MMP-derived metabolite of citrullinated vimentin, has been associated with disease activity and radiographic progression in AS [21].

The aim of this study was to investigate a panel of ECM biomarkers to link ECM turnover with disease activity measures to identify candidate serum markers of tissue homeostasis and disturbance associated with disease activity. Such markers could potentially enable better monitoring of disease development in patients with axSpA. Therefore, we investigated the association of the ECM metabolite biomarkers PRO-C1, PRO-C3, PRO-C4, PRO-C6, C3M, C4M, C6M, C7M, C10C, CRPM, EL-NE, and VICM with disease activity in patients with axSpA, as measured clinically and by MRI, and their relationship with the treatment response to TNF inhibitory therapy for 46 weeks.

## Methods

### Subjects

The BIOSPA study has been described previously [22, 23]. Briefly, 60 TNFα inhibitor-naïve patients with axSpA initiating TNF-α inhibitor therapy were recruited from nine Danish rheumatology departments. Clinical and biochemical assessments were performed at baseline, and patients were followed for 46 weeks. Seven patients discontinued before week 22, and another six patients discontinued before week 46 due to side effects or lack of efficacy. Clinical response was defined as a reduction in the Bath AS Disease Activity Score (BASDAI) of 50% or 20 mm (on a VAS scale of 0–100 mm) at week 22 [24]. Since the study was performed before ASDAS was introduced, this was calculated post hoc. ASDAS responses were defined as follows: no clinically important improvement (ΔASDAS<1.1), clinically important improvement (1.1≤ΔASDAS<2.0), and major improvement in ASDAS (ΔASDAS≥2.0) [25]. The Spondyloarthritis Research Consortium of Canada (SPARCC) MRI Spine and Sacroiliac Joint Inflammation score, which quantitatively assesses bone marrow edema of the spine and sacroiliac joint, was evaluated by an experienced reader (UW )[22]. Serum was taken according to standard operating procedures and stored at −80°C prior to biomarker measurement [26].

### Biomarker analysis

Biomarkers were assessed in 55 patients with available blood samples. Twelve different ECM metabolite biomarkers were assessed by competitive ELISAs developed by Nordic Bioscience. The assays measure type I collagen (PRO-C1), type III collagen (PRO-C3), type IV collagen (PRO-C4), and type VI collagen (PRO-C6) formation; MMP-generated type III collagen (C3M), type IV collagen (C4M), type VI collagen (C6M), and type VII collagen (C7M) degradation; along with Cathepsin K-generated type X collagen, MMP-mediated metabolite of C-reactive protein (CRPM), neutrophil elastase-degraded elastin (*EL*-*NE*), and citrullinated vimentin (VICM). All nine biomarkers were measured in serum or EDTA plasma at baseline (*n*=55), week 2, week 22, and week 46 after treatment with TNF-inhibitory therapy (infliximab, etanercept, or adalimumab). The assays were previously developed and technically validated [15, 17, 27–34]. The inter- and intra-assay coefficients of variation were <15% and <10%, respectively. Samples below the lower limit of quantification (LLOQ) were assigned the value of LLOQ.

### Statistical analysis

Baseline characteristics were presented as numbers (frequency) and percentages for categorical variables and as medians (interquartile range) for continuous variables. Differences between the biomarker levels at the four timepoints were calculated using a repeated measures analysis of variance (ANOVA). Comparison of baseline variables was performed with the Mann–Whitney test, while correlations were performed with Spearman’s rank correlation coefficient. Before the analyses, a total SPARCC MRI Spine and Sacroiliac Joint Inflammation score was calculated as the sum of the SPARCC MRI Spine Inflammation score and the SPARCC MRI Sacroiliac Joint Inflammation score. Analysis was performed in MedCalc (version 14.8.1) and GraphPad Prism (version 8) software. A p-value below 0.05 was considered significant.

## Results

### Baseline demographics

Clinical and demographic characteristics at baseline are shown in Table 1. The patients in the study were mostly (82%) male. Furthermore, 84% of the patients were HLA-B27 positive, and the median age was 40 years (inter quartile range (IQR): 32–48). The patients had a median disease duration of 5.5 years (IQR: 2–10) and a median baseline ASDAS of 3.9 (IQR: 3.0–4.5). At baseline, weak correlations were observed between PRO-C1, PRO-C4, VICM, and ASDAS (*ρ*=−0.286, *p*=0.038; *ρ*=0.315, *p*=0.022; and *ρ*=0.327, *p*=0.017, respectively), while moderate correlations were observed for C6M versus ASDAS (*ρ*=0.538, *p*<0.001) and CRP versus ASDAS (*ρ*=0.654, *p*<0.0001). At baseline, no biomarkers were correlated with BASDAI or swollen joint count.

**Table 1:** Demographics and baseline clinical characteristics

	Patients (***n***=55)
**HLA-B27 positive (%)**	53 (96%)
**Gender (male/female) (%)**	45 (82%)/10 (18%)
**Age, years (IQR)**	40 (32–48)
**Disease duration (IQR)**	5.5 (2.0–10.0)
**Baseline NSAID use (%)**	47 (85%)
**Infliximab/etanercept/adalimumab**	37 (67%)/12 (22%)/6 (11%)
**ASDAS**	3.9 (3.0–4.5)
**BASDAI (0–100)**	55.0 (44.0–72.8)
**Swollen joint count (0–28)**	0 (0-8)
**Serum CRP (mg/L)**	27.6 (1.2–162.0)
**Patients with a SpA feature, n (%)**	24 (44%) patients with 1 SpA feature 4 (7%) patients with 2 SpA features 2 (4%) patients with 3 SpA features
**Peripheral arthritis (%)**	12 (22%)
**Psoriasis (%)**	12 (22%)
**History of anterior uveitis (%)**	11 (20%)
**History of inflammatory bowel disease (%)**	3 (5%)

Values are given as numbers (frequency) and percentages for categorical variables and as medians (interquartile range (IQR)) for continuous variables

Information on the number (%) of patients with a SpA feature and the number (%) of a particular SpA feature were based on patient history and clinical examination at study inclusion

*HLA-B27* human leucocyte antigen B27, *CRP* C-reactive protein, *ASDAS* Ankylosing Spondylitis Disease Activity Score, *BASDAI* Bath ankylosing spondylitis disease activity index, *NSAID* nonsteroidal anti-inflammatory drugs, *SpA* spondyloarthritis

After 22 weeks of treatment, 10 patients were classified as showing clinically important improvement in ASDAS, while 23 patients were classified as showing major improvement in ASDAS. After 46 weeks of treatment, 13 and 17 patients, respectively were classified as having achieved a clinically important and a major improvement in ASDAS, respectively.

### Changes in PRO-C1, C6M, EL-NE, VICM, and CRP during TNF-α inhibitory therapy

**Fig. 1:**
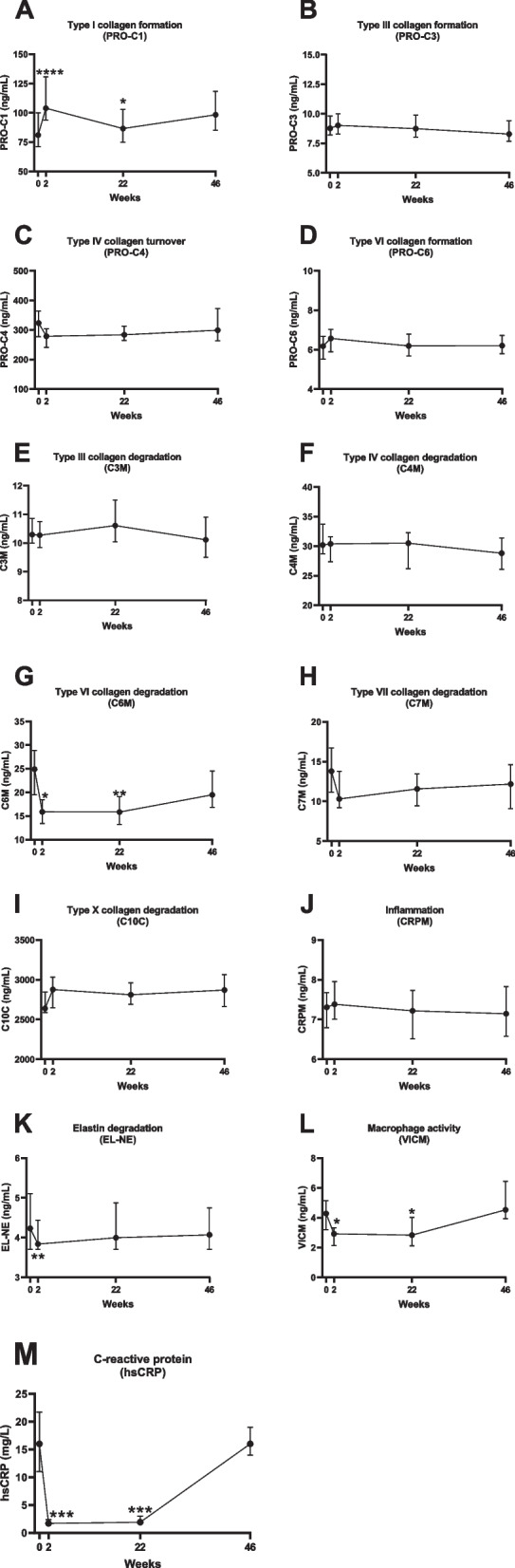
Time course of biomarkers in response to TNF-α inhibitor therapy. **A** Type I collagen formation, PRO-C1; **B** type III collagen formation, PRO-C3; **C** type IV collagen turnover, PRO-C4; **D** type VI collagen formation, PRO-C6; **E** type III collagen degradation, C3M; **F** type IV collagen degradation, C4M; **G** type VI collagen degradation, C6M; **H** type VII collagen degradation, C7M; **I** type X collagen degradation, C10C; **J** C-reactive metabolite, CRPM; **K** elastin degradation, EL-NE; **L** citrullination and degradation of vimentin, VICM; and **M** C-reactive protein, CRP. Time course is shown as median ± 95% CI. *p<0.05 **p<0.01, ***p<0.001, ****p<0.0001

Compared to baseline, circulating levels of PRO-C1 significantly increased after 2 and 22 weeks of treatment (*p*<0.0001 and *p*=0.037, respectively; Fig. 1A), whereas C6M levels significantly decreased after 2 and 22 weeks of treatment (*p*=0.001 and *p*=0.002, respectively; Fig. 1G). Compared to baseline, EL-NE levels were significantly decreased after 2 weeks of treatment (*p*=0.0008; Fig. 1K). VICM levels decreased after 2 and 22 weeks of treatment compared to baseline (*p*=0.0163 and *p*=0.0374, respectively; Fig. 1L). CRP levels decreased after 2 and 22 weeks of treatment compared to baseline (both *p*=0.0001; Fig. 1M). In the other biomarkers, no significant changes were observed from baseline to weeks 2, 22, and 46. In comparison, there was a significant decrease in the ASDAS and BASDAI scores from baseline to 2, 22, and 46 weeks (both *p*<0.0001) as well as a decrease in SPARCC scores from baseline to 22 weeks (*p*=0.005) and 46 weeks (*p*=0.0005).

### Association of PRO-C1, PRO-C3, C6M, VICM, and CRP with improvement in ASDAS

After 22 weeks, 17 individuals did not achieve a clinically important or major improvement in ASDAS (ΔASDAS <1.1), while 10 patients achieved a clinical important improvement in ASDAS (ΔASDAS ≥1.1) and 23 individuals achieved a major improvement (ΔASDAS ≥2.0). After 46 weeks of treatment, 11 patients did not achieve a clinically important or major improvement in ASDAS, while 13 and 17 patients achieved clinically important and major improvement in ASDAS, respectively. Levels of PRO-C1, PRO-C3, C6M, VICM and CRP were all associated with clinically and major improvement after 22 weeks (*p*=0.006, *p*=0.008, *p*<0.001, <0.001, and *p*<0.001, respectively; Table 2), while C6M, VICM, and CRP were the only three biomarkers associated with a change in ASDAS score after 46 weeks (*p*=0.002, *p*=0.044, and *p*<0.001, respectively; Table 2). There was no association between the other biomarkers and improvement in ASDAS scores at weeks 22 and 46.

**Table 2:** Changes in collagen biomarker levels and ASDAS improvement categories from baseline to weeks 22 and 46, respectively

Biomarker	Baseline to week 22				Baseline to week 46			
	No improvement (***n***=17)	Clinically important improvement (***n***=10)	Major improvement (***n***=23)	P-value	No improvement (***n***=11)	Clinically important improvement (***n***=13)	Major improvement (***n***=17)	***P*** value
**PRO-C1** *Median (IQR)*	0.1 (− 32.8, 7.8)	7.6 (− 3.5, 16.0)	29.5 (9.2, 44.7)	**0.006**	0.1 (− 32.6, 8.8)	28.4 (5.9, 47.3)	33.8 (6.5, 52.3)	**0.006**
**PRO-C3** *Median (IQR)*	− 13.1 (− 32.4, − 3.2)	3.2 (− 3.9, 9.0)	12.6 (− 1.4, 32.9)	**0.008**	6.3 (− 3.9, 25.5)	− 3.9 (− 16.0, 13.2)	5.7 (− 2.2, 29.0)	0.573
**PRO-C4** *Median (IQR)*	10.2 (− 12.7, 20.3)	− 14.0 (− 24.3, 10. 6)	− 15.6 (− 37.5, 0.9)	0.114	3.6 (− 22.5, 15.7)	17.7 (− 15.6, 22.4)	− 24.2 (− 43.0, − 8.4)	**0.048**
**PRO-C6** *Median (IQR)*	− 6.7 (− 13.9, 0.0)	0.3 (− 13.2, 10.7)	7.6 (− 6.4, 23.6)	0.063	0.3 (− 2.5, 17.5)	7.6 (− 12.3, 17.3)	6.2 (− 6.9, 21.4)	0.915
**C3M** *Median (IQR)*	7.9 (− 3.1, 18.9)	20.4 (11.3, 22.2)	2.9 (− 14.4, 11.8)	0.152	11.1 (0.7, 19.7)	8.3 (− 3.1, 20.9)	2.9 (− 14.3, 9.8)	0.318
**C4M** *Median (IQR)*	− 7.2 (− 14.8, 8.8)	5.0 (− 22.3, 16.4)	− 6.8 (− 11.4, 6.9)	0.899	− 2.0 (− 9.1, 5.9)	− 11.5 (− 30.8, − 7.7)	− 0.3 (− 9.7, 11.1)	0.153
**C6M** *Median (IQR)*	12.4 (− 34.8, 22.3)	− 20.2 (− 29.8, 19.9)	− 54.3 (− 70.8, − 30.1)	**< 0.001**	12.0 (− 25.4, 37.1)	− 1.9 (− 43.2, 22.3)	− 62.1 (− 73.7, − 30.0)	**0.007**
**C7M** *Median (IQR)*	− 26.9 (− 31.1, 35.3)	− 33.5 (− 64.5, − 17.1)	− 32.2 (− 55.4, 0.0)	0.192	− 29.1 (− 43.6, 41.0)	− 26.9 (− 39.0, 0.0)	− 46.6 (− 56.9, − 4.5)	0.447
**C10C** *Median (IQR)*	2.8 (− 9.3, 17.6)	17.4 (5.3, 23.9)	2.1 (− 6.1, 15.5)	0.216	19.7 (10.1, 22.7)	0.5 (− 9.9, 12.4)	4.0 (− 4.7, 14.5)	0.165
**CRPM** *Median (IQR)*	− 2.3 (− 8.6, 5.1)	− 1.3 (− 4.8, 20.5)	2.5 (− 16.3, 15.1)	0.541	5.1 (− 0.8, 23.0)	− 5.1 (− 9.1, 1.4)	− 1.1 (− 25.4, 10.4)	0.248
**EL-NE** *Median (IQR)*	0.0 (− 17.2, 9.1)	1.2 (0.0, 24.9)	0.0 (− 33.1, 0.0)	0.108	0.0 (− 8.6, 1.4)	0.0 (− 30.0, 29.3)	0.0 (− 31.7, 0.0)	0.414
**VICM** *Median (IQR)*	29.1 (− 17.3, 107.4)	− 72.1 (− 80.1, − 58.0)	− 43.3 (− 65.3, − 0.7)	**< 0.001**	0.0 (− 35.8, 47.8)	− 34.7 (− 64.9, − 18.8)	− 63.8 (− 69.7, − 37.9)	**0.044**
**CRP** ***Median (IQR)***	− 1.7 (− 4.93 to − 0.63)	− 9.2 (− 24.80–5.30)	− 24.9 (− 61.40 to − 12.70)	**<0.001**	12.3 (8.00–27.68)	− 1.50 (9.500–5.58)	− 7.00 (65.25–1.25)	**<0.001**

*PRO-C1* type I collagen formation, *PRO-C3* type III collagen formation, *PRO-C4* type IV collagen turnover, *PRO-C6* type VI collagen formation, *C3M* degradation of type III collagen, *C4M* degradation of type IV collagen, *C6M* degradation of type VI collagen, *C7M* degradation of type *VII* collagen, *C10C* degradation of type X collagen, *CRPM* degradation of C-reactive protein, *EL-NE* degradation of elastin, *VICM* degraded and citrullinated vimentin, *CRP* C-reactive protein, *IQR* inter quartile range

### Association of PRO-C1 and C6M with BASDAI response

Compared to non-responders, responders had significantly higher levels of PRO-C1 after 22 weeks, while C6M levels were significantly lower at baseline for patients who were responding to TNF-a inhibitory therapy compared to non-responders (*p*=0.020 and *p*=0.049, respectively; Table 3). No association was found for responders after 46 weeks. There was no association between the other biomarkers and BASDAI response at weeks 22 and 46.

**Table 3:** Response to TNF-α treatment. Percent changes of biomarkers from baseline to weeks 22 and 46 in patients with and without a BASDAI response, respectively

Biomarker	Week 22 response			Week 46 response		
	Non-responders ***n***=19	Responders ***n***=36	***p***-value	Non-responders ***n***=12	Responders ***n***=37	***p***-value
**PRO-C1** *Median (IQR)*	− 3.1 (− 33.2, 13.9)	14.2 (5.1, 36.1)	**0.020**	6.2 (− 15.4, 24.1)	12.8 (1.5, 36.1)	0.432
**PRO-C3** *Median (IQR)*	− 6.0 (− 27.6, 8.4)	4.0 (− 11.0, 28.6)	0.186	− 1.4 (− 17.0, 25.5)	6.5 (− 11.0, 28.6)	0.552
**PRO-C4** *Median (IQR)*	− 12.7 (− 28.1, 28.9)	− 8.1 (− 25.3, 17.8)	0.832	− 11.9 (− 29.2, 10.1)	− 8.4 (− 25.7, 22.4)	0.710
**PRO-C6** *Median (IQR)*	− 2.5 (− 13.8, 8.7)	− 0.4 (− 12.1, 19.1)	0.349	− 0.4 (− 16.0, 19.9)	0.2 (− 12.4, 14.2)	0.868
**C3M** *Median (IQR)*	15.3 (− 7.1, 21.9)	4.5 (− 9.2, 15.0)	0.331	5.7 (− 8.9, 20.4)	6.0 (− 9.5, 16.4)	0.739
**C4M** *Median (IQR)*	2.7 (− 14.2, 15.1)	− 6.8 (− 13.7, 5.5)	0.517	− 11.3 (− 14.8, 2.4)	− 6.4 (− 12.2, 6.7)	0.421
**C6M** *Median (IQR)*	− 3.7 (− 39.0, 32.1)	− 32.0 (− 63.7, 4.5)	**0.049**	− 9.9 (− 56.3, 48.2)	− 30.6 (− 53.9, 17.1)	0.485
**C7M** *Median (IQR)*	− 29.1 (− 42.5, 34.8)	− 28.9 (− 54.5, 8.4)	0.737	− 22.2 (− 56.1, 39.6)	− 30.0 (− 51.5, 0.0)	0.642
**C10C** *Median (IQR)*	9.2 (− 9.2, 21.0)	2.4 (− 5.5, 15.0)	0.873	13.4 (− 4.3, 22.4)	2.1 (− 8.7, 14.5)	0.403
**CRPM** *Median (IQR)*	− 1.8 (− 11.0, 6.8)	− 3.6 (− 14.7, 16.3)	0.854	1.5 (− 8.2, 10.7)	− 4.2 (− 13.1, 10.6)	0.490
**EL-NE** *Median (IQR)*	0.0 (0.0, 11.6)	0.0 (− 31.9, 0.9)	0.222	0.2 (0.0, 23.1)	0.0 (− 32.6, 8.0)	0.212
**VICM** *Median (IQR)*	− 17.3 (− 63.6, 49.1)	− 36.3 (− 66.0, 9.4)	0.559	− 16.1 (− 70.3, 27.8)	− 37.9 (− 64.9, 42.3)	0.926
**CRP** ***Median (IQR)***	− 56.7 (− 82.66–26.78)	− 83.64 (− 94.10–58.75)	0.086	183.3 (− 33.81–458.11)	− 9.5 (60.34–120.71)	0.456

*PRO-C1* type I collagen formation, *PRO-C3* type III collagen formation, *PRO-C4* type IV collagen turnover, *PRO-C6* type VI collagen formation, *C3M* degradation of type III collagen, *C4M* degradation of type IV collagen, *C6M* degradation of type VI collagen, *C7M* degradation of type VII collagen, *C10C* degradation of type X collagen, *CRPM* degradation of C-reactive protein, *EL-NE* degradation of elastin, *VICM* degraded and citrullinated vimentin; *IQR* inter quartile range

### Association of PRO-C1, PRO-C4, C6M, and C7M with SPARCC MRI sacroiliac joint and spine inflammation scores

Baseline and week 22 levels of PRO-C4 and C6M were mildly to moderately correlated with the total SPARCC MRI Spine and Sacroiliac Joint Inflammation score (PRO-C4: *ρ*=0.279, *p*=0.043 and *ρ*=0.317, *p*=0.021, respectively, and C6M: *ρ* =0.496, *p*=0.0002 and ρ =0.297, *p*=0.031, respectively; Table 4). Week 22 and week 46 levels of PRO-C1 and C7M were mildly correlated with the total SPARCC Spine and Sacroiliac Joint Inflammation score (PRO-C1: *ρ*=− 0.318, *p*=0.021 and *p*=-0.285, *p*=0.05, respectively; C7M: *ρ*=0.379, *p*=0.005 and *ρ*=0.319, *p*=0.029, respectively; Table 4). No correlations were found between CRP and SPARCC at baseline (*ρ*=− 0.205, *p*=0.153), week 22 (*ρ*=0.263, *p*=0.057), or week 46 (*ρ*=− 0.127, *p*=0.430) or the other ECM biomarkers investigated in the study.

**Table 4:** Correlation between the biomarkers, CRP, and total SPARCC score. Spearman’s correlation was performed between extracellular matrix metabolites and MRI scores. Spearman’s rho (*ρ*) and *p*-values are shown

Biomarker	SPARCC, baseline	SPARCC, week 22	SPARC, week 46
PRO-C1			
*ρ*	0.086	**− 0.318**	**− 0.285**
*p*-value	0.542	**0.021**	**0.050**
PRO-C3			
*ρ*	**−** 0.012	**−** 0.110	0.135
*p*-value	0.933	0.436	0.360
PRO-C4			
*ρ*	**0.279**	**0.317**	0.216
*p*-value	**0.043**	**0.021**	0.144
PRO-C6			
*ρ*	0.031	**−** 0.187	**−** 0.110
*p*-value	0.825	0.184	0.457
C3M			
*ρ*	**−** 0.006	0.222	0.235
*p*-value	0.966	0.113	0.108
C4M			
*ρ*	**−** 0.070	0.081	0.281
*p*-value	0.623	0.568	0.056
C6M			
*ρ*	**0.496**	**0.297**	0.034
*p*-value	**0.0002**	**0.031**	0.818
C7M			
*ρ*	0.130	**0.379**	**0.319**
*p*-value	0.355	**0.005**	**0.029**
C10C			
*ρ*	0.018	0.112	0.170
*p*-value	0.897	0.425	0.255
CRPM			
*ρ*	**−** 0.169	0.152	0.170
*p*-value	0.227	0.283	0.247
EL-NE			
*ρ*	**−** 0.088	**−** 0.011	0.228
*p*-value	0.530	0.935	0.119
VICM			
*ρ*	0.158	**−** 0.153	**−** 0.228
*p*-value	0.258	0.273	0.123
CRP			
*ρ*	**−** 0.205	0.263	**−** 0.228
*p*-value	0.153	0.057	0.123

## Discussion

We evaluated changes in ECM metabolites (PRO-C1, PRO-C3, PRO-C4, PRO-C6, C3M, C4M, C6M, C7M, C10C, CRPM, EL-NE, and VICM) as potential biomarkers of disease activity and treatment response in patients with axSpA initiating treatment with TNF-α inhibitor and followed for 46 weeks. The main findings were as follows: (i) PRO-C1 was increased at weeks 2 and 22 after the initiation of TNF-inhibitory therapy, while C6M, EL-NE, and VICM were decreased at week 2, and both C6M and VICM remained decreased after week 22; (ii) PRO-C1, PRO-C3, C6M, and VICM were associated with a clinical improvement in the ASDAS score; (iii) PRO-C1 and C6M were associated with a BASDAI response to TNF-inhibitory therapy; and (iv) PRO-C4 and C6M were associated with total SPARCC MRI Spine and Sacroiliac Joint scores at baseline.

In the past decade, progress has been made on the development of biologics for treating axSpA. However, reliable and commonly available tools to guide treatment decisions are still needed. Candidate biomarkers reflecting disease activity, response to treatment, and structural progression may help close this gap [3, 35–37]. An essential property for an ECM metabolite biomarker in axSpA is a relationship with MRI inflammation and treatment response scores, thus potentially reflecting the impact of disease activity on the tissue level. In this study, we demonstrated a correlation between ECM metabolites and the total SPARCC MRI Spine and Sacroiliac Joint Inflammation score at baseline as well as associations with measures of clinical improvement and response to treatment, indicating that these metabolites may represent valuable biomarkers of disease activity in axSpA. In this study, C6M was reduced by TNF-inhibitory therapy, associated with clinically important and major improvement in ASDAS disease activity at weeks 22 and 46, and it moderately correlated with the total SPARCC MRI Spine and Sacroiliac Joint Inflammation score, and differentiated responders from non-responders after 22 weeks. Type VI collagen (COL6) is an ECM protein located in the interface between the basement membrane and interstitial matrix, where it binds to other ECM proteins and supports cell-to-cell interactions [38–41]. C6M is a metabolite of COL6 that is released from the inflamed tissue and can be measured in serum as a biomarker of connective tissue remodeling [27]. Previously, C6M has been associated with AS in a cross-sectional setting, where it was used to distinguish between healthy controls and AS patients, and it has been suggested as a candidate marker for monitoring disease activity in interventional studies [14, 42]. C6M separated AS patients from healthy individuals with an area under curve (AUC) of 0.78, but it was not correlated with radiographic progression in the spine (mSASSS). In another radiographic axSpA (r-axSpA) clinical study, C6M was shown to be highly correlated with CRP but not mSASSS [43]. However, the associations between C6M and the composite measure of disease activity for patients with axSpA (ASDAS) have not been studied previously. While chronic inflammation is a hallmark of axSpA, the nature of inflammation is not fully elucidated. Inflammation typically accelerates the turnover of collagens [44], and thus it was expected that C6M would be associated with MRI inflammation and CRP at baseline, which was demonstrated in this study. In addition, C6M is a marker of connective tissue breakdown, whereas CRP is a non-specific acute phase reactant, which are increased in many conditions. The potential of C6M as a biomarker of treatment response has also recently been shown in patients with psoriatic arthritis (PsA) [45].

Another interesting biomarker in this study is PRO-C1, which increased 2 and 22 weeks following TNF-inhibitory therapy and showed associations with disease activity and response to treatment. Type I collagen is the major collagen in the human body and the major constituent of bone. PRO-C1, also known as PINP, can be used to measure bone formation. The bone phenotype in axSpA is a mix of processes involving bone loss and bone formation. Bone loss (osteoporosis and bone erosion) is caused by increased osteoclast activity, although the link between TNF-inhibitory therapy and osteoclast activity has been debated; however, most findings indicate that TNF-inhibitory therapy leads to the inhibition of osteoblast differentiation [46–48]. An observational study of AS patients treated with TNF inhibitor reported that structural progression continued after the initiation of therapy, and retardation of structural damage was reported only after 4 years of treatment [49]. This may support the increase of PRO-C1 after 2 and 22 weeks. A third biomarker, VICM, was decreased after 2 and 22 weeks of TNF-inhibitor therapy and was also associated with clinically important and major improvements after 22 and 46 weeks. Vimentin is a type III intermediate filament protein that is expressed by various cells as a constituent of the cytoskeleton. It is secreted by macrophages and known to be citrullinated intercellularly by PAD enzymes [50]. VICM measures a citrullinated and MMP-mediated fragment of vimentin, and it has been reported to be prognostic for radiographic progression in AS [21], whereas VICM has not been shown to be modulated by TNF-inhibitory therapy [21].

## Conclusion

In conclusion, the study demonstrates that extracellular matrix metabolites are associated with clinically important and major improvement in ASDAS, MRI inflammation, and response to TNF-inhibitory treatment in patients with axSpA. Selected biomarkers reflecting bone formation (PRO-C1), soft tissue degradation (C6M), and macrophage activity (VICM) are candidate soluble tissue turnover biomarkers of disease activity in axSpA.

## Data Availability

All data from this study are included in this article. Data are kept on file and are not publicly available due to privacy data legislation. Requests can be directed to the corresponding author.

## Abbreviations

ASDAS: Ankylosing Spondylitis Disease Activity Score
axSpA: Axial spondyloarthritis
BASDAI: Bath AS Disease Activity Score
BIOSPA: Biomarkers in Spondyloarthritis (Prospective observational cohort study)
BME: Bone marrow edema
C3M: Degradation of type III collagen
C4M: Degradation of type IV collagen
C6M: Degradation of type VI collagen
C7M: Degradation of type VII collagen
C10C: Degradation of type X collagen
CRP: C-reactive protein
CRPM: Metabolite of C-reactive protein
ECM: Extracellular matrix
EDTA: Ethylenediamine tetraacetic acid
EL-NE: Degradation of elastin
HLA-B27: Human leukocyte antigen B2
MMP: Matrix metalloproteinase
MRI: Magnetic resonance imaging
MTA: Methotrexate
mSASSS: Modified Stoke Ankylosing Spondylitis Spine Score
PAD: Peptidylarginine deiminases
PRO-C1: Formation of type I collagen
PRO-C3: Formation of type III collagen
PRO-C6: Formation of type VI collagen
PRO-C4: Turnover of type IV collagen, 7S domain
SPARCC: The Spondyloarthritis Research Consortium of Canada
TNF: Tumor necrosis factor
VICM: Citrullinated and degraded vimentin

## References

[1] Rudwaleit M, Van Der Heijde D, Landewé R, Akkoc N, Brandt J, Chou CT (2011). The Assessment of SpondyloArthritis international Society classification criteria for peripheral spondyloarthritis and for spondyloarthritis in general. Ann Rheum Dis.

[2] Braun J, Bollow M, Remlinger G, Eggens U, Rudwaleit M, Distler A (1998). Prevalence of spondylarthropathies in HLA-B27 positive and negative blood donors. Arthritis Rheum.

[3] Maksymowych WP (2015). Biomarkers in axial spondyloarthritis. Curr Opin Rheumatol.

[4] Landewé R, van Tubergen A (2015). Clinical Tools to Assess and Monitor Spondyloarthritis. Curr Rheumatol Rep.

[5] Proft F, Muche B, Spiller L, Rios Rodriguez V, Rademacher J, Weber AK (2020). Performance of the Ankylosing Spondylitis Disease Activity Score based on a quick quantitative C-reactive protein assay in patients with axial spondyloarthritis. Jt Bone Spine.

[6] Lukas C, Cyteval C, Dougados M, Weber U (2018). MRI for diagnosis of axial spondyloarthritis: Major advance with critical limitations “Not everything that glisters is gold (standard).”. RMD Open.

[7] Moz S, Aita A, Basso D, Ramonda R, Plebani M, Punzi L (2017). Spondyloarthritis: Matrix metalloproteinasesas biomarkers of pathogenesis and response to tumor necrosis factor (TNF) inhibitors. Int J Mol Sci.

[8] Karsdal MA, Henriksen K, Leeming DJ, Mitchell P, Duffin K, Barascuk N (2009). Biochemical markers and the FDA Critical Path: how biomarkers may contribute to the understanding of pathophysiology and provide unique and necessary tools for drug development. Biomarkers..

[9] Mienaltowski M, Birk D (2014). Structure, physiology, and biochemistry of collagens. Adv Exp Med Biol.

[10] Holm Nielsen S, Sardar S, Siebuhr A, Schlemmer A, Schmidt E, Bay-Jensen A (2021). Effect of n-3 PUFA on extracellular matrix protein turnover in patients with psoriatic arthritis: a randomized, double-blind, placebo-controlled trial. Rheumatol Int.

[11] Dobrota R, Jordan S, Juhl P, Maurer B, Wildi L, Bay-Jensen AC (2021). Circulating collagen neo-epitopes and their role in the prediction of fibrosis in patients with systemic sclerosis: a multicentre cohort study. Lancet Rheumatol.

[12] van Haaften WT, Mortensen JH, Dige AK, Grønbæk H, Hvas CL, Bay-Jensen AC (2020). Serological Biomarkers of Tissue Turnover Identify Responders to Anti-TNF Therapy in Crohn’s Disease: A Pilot Study. Clin Transl Gastroenterol.

[13] Bay-Jensen AC, Madsen S, Gehring K, Musa K, Karsdal M. Rheumatoid arthritis is driven not only by inflammation but also fibrogenesis. Ann Rheum Dis. 2021;8(Supp. 1):AB0067.

[14] Bay-Jensen AC, Leeming DJ, Kleyer A, Veidal SS, Schett G, Karsdal MA (2012). Ankylosing spondylitis is characterized by an increased turnover of several different metalloproteinase-derived collagen species: a cross-sectional study. Rheumatol Int.

[15] Sand JMB, Lamy P, Juhl P, Siebuhr AS, Iversen LV, Nawrocki A (2018). Development of a Neo-Epitope Specific Assay for Serological Assessment of Type VII Collagen Turnover and Its Relevance in Fibroproliferative Disorders. Assay Drug Dev Technol.

[16] Gudmann NS, Munk HL, Christensen AF, Ejstrup L, Sørensen GL, Loft AG (2016). Chondrocyte activity is increased in psoriatic arthritis and axial spondyloarthritis. Arthritis Res Ther.

[17] He Y, Siebuhr AS, Brandt-hansen NU, Wang J, Su D, Zheng Q (2014). Type X collagen levels are elevated in serum from human osteoarthritis patients and associated with biomarkers of cartilage degradation and inflammation. BMC Musculoskelet Disord.

[18] Mortensen JH, Manon-Jensen T, Jensen MD, Hägglund P, Klinge LG, Kjeldsen J (2017). Ulcerative colitis, Crohn’s disease, and irritable bowel syndrome have different profiles of extracellular matrix turnover, which also reflects disease activity in Crohn’s disease. PLoS One.

[19] Siebuhr AS, Hušaková M, Forejtová S, Zegzulková K, Tomčik M, Urbanová M (2019). Metabolites of C-reactive protein and vimentin are associated with disease activity of axial spondyloarthritis. Clin Exp Rheumatol.

[20] Skjøt-Arkil H, Schett G, Zhang C, Larsen DV, Wang Y, Zheng Q (2012). Investigation of two novel biochemical markers of inflammation, matrix metalloproteinase and cathepsin generated fragments of C-reactive protein, in patients with ankylosing spondylitis. Clin Exp Rheumatol.

[21] Bay-jensen AC, Karsdal MA, Vassiliadis E, Wichuk S, Marcher-mikkelsen K, Lories R (2013). Circulating Citrullinated Vimentin Fragments Reflect Disease Burden in Ankylosing Spondylitis and Have Prognostic Capacity for Radiographic Progression. Arthritis Rheum.

[22] Pedersen SJ, Sørensen IJ, Hermann KGA, Madsen OR, Tvede N, Hansen MS (2010). Responsiveness of the Ankylosing Spondylitis Disease Activity Score (ASDAS) and clinical and MRI measures of disease activity in a 1-year follow-up study of patients with axial spondyloarthritis treated with tumour necrosis factor α inhibitors. Ann Rheum Dis.

[23] Pedersen SJ, Sørensen IJ, Garnero P, Johansen JS, Madsen OR, Tvede N (2011). ASDAS, BASDAI and different treatment responses and their relation to biomarkers of inflammation, cartilage and bone turnover in patients with axial spondyloarthritis treated with TNFα inhibitors. Ann Rheum Dis.

[24] Braun J, Baraliakos X, Golder W, Brandt J, Rudwaleit M, Listing J (2003). Magnetic resonance imaging examinations of the spine in patients with ankylosing spondylitis, before and after successful therapy with infliximab: evaluation of a new scoring system. Arthritis Rheum.

[25] MacHado P, Landewé R, Lie E, Kvien TK, Braun J, Baker D (2011). Ankylosing Spondylitis Disease Activity Score (ASDAS): Defining cut-off values for disease activity states and improvement scores. Ann Rheum Dis.

[26] Tuck MK, Chan DW, Chia D, Godwin AK, Grizzle WE, Krueger KE (2009). Standard Operating Procedures for Serum and Plasma Collection: Early Detection Research Network Consensus Statement Standard Operating Procedure Integration Working Group. J Proteome Res.

[27] Veidal SS, Karsdal MA, Vassiliadis E, Nawrocki A, Larsen MR, Nguyen QHT (2011). MMP mediated degradation of type VI collagen is highly associated with liver fibrosis--identification and validation of a novel biochemical marker assay. PLoS One.

[28] Bay-Jensen AC, Liu Q, Byrjalsen I, Li Y, Wang J, Pedersen C (2011). Enzyme-linked immunosorbent assay (ELISAs) for metalloproteinase derived type II collagen neoepitope, CIIM-Increased serum CIIM in subjects with severe radiographic osteoarthritis. Clin Biochem.

[29] Barascuk N, Veidal SS, Larsen L, Larsen DV, Larsen MR, Wang J (2010). A novel assay for extracellular matrix remodeling associated with liver fibrosis: An enzyme-linked immunosorbent assay (ELISA) for a MMP-9 proteolytically revealed neo-epitope of type III collagen. Clin Biochem.

[30] Nielsen MJ, Karsdal MA, Kazankov K, Grønbæk H, Krag A, Leeming DJ (2016). Fibrosis is not just fibrosis – basement membrane modelling and collagen metabolism differs between hepatitis B- and C-induced injury. Aliment Pharmacol Ther.

[31] Nielsen MJ, Nedergaard AF, Sun S, Veidal SS, Larsen L, Zheng Q (2013). The neo-epitope specific PRO-C3 ELISA measures true formation of type III collagen associated with liver and muscle parameters. Am J Transl Res.

[32] Leeming DJ, Larsen DV, Zhang C, Hi Y, Veidal SS, Nielsen RH (2010). Enzyme-linked immunosorbent serum assays (ELISAs) for rat and human N-terminal pro-peptide of collagen type I (PINP)--assessment of corresponding epitopes. Clin Biochem.

[33] Sun S, Karsdal MA, Bay-jensen AC, Sørensen MG, Zheng Q, Dziegiel MH (2013). The development and characterization of an ELISA speci fi cally detecting the active form of cathepsin K. Clin Biochem.

[34] Sand JM, Larsen L, Hogaboam C, Martinez F, Han M, Røssel Larsen M (2013). MMP mediated degradation of type IV collagen alpha 1 and alpha 3 chains reflects basement membrane remodeling in experimental and clinical fibrosis--validation of two novel biomarker assays. PLoS One.

[35] Maksymowych WP. Biomarkers for diagnosis of axial spondyloarthritis, disease activity, prognosis, and prediction of response to therapy. Front Immunol. 2019:305–25. 10.3389/fimmu.2019.00305 PMC6416369 30899255

[36] Schett G. Bone formation versus bone: Resorption in ankylosing spondylitis. Adv Exp Med Biol. 2009;114–21. 10.1007/978-1-4419-0298-6_8 19731624

[37] De Vlam K. Soluble and tissue biomarkers in ankylosing spondylitis. Best Pract Res Clin Rheumatol. 2010:671–82. 10.1016/j.berh.2010.05.009 21035087

[38] Kuo H-J, Maslen CL, Keene DR, Glanville RW (1997). Type VI Collagen Anchors Endothelial Basement Membranes by Interacting with Type IV Collagen* Downloaded from. J Biol Chem.

[39] Cescon M, Gattazzo F, Chen P, Bonaldo P (2015). Collagen VI at a glance. J Cell Sci.

[40] Groulx JF, Gagné D, Benoit YD, Martel D, Basora N, Beaulieu JF (2011). Collagen VI is a basement membrane component that regulates epithelial cell-fibronectin interactions. Matrix Biol.

[41] Karsdal MA, Krarup H, JMB S, Christensen PB, Gerstoft J, Leeming DJ (2014). The efficacy of biomarkers in chronic fibroproliferative diseases - Early diagnosis and prognosis, with liver fibrosis as an exemplar. Aliment Pharmacol Ther.

[42] Siebuhr AS, Bay-jensen AC, Leeming DJ, Plat A, Byrjalsen I, Christiansen C (2013). Serological identification of fast progressors of structural damage with rheumatoid arthritis. Arthritis Res Ther.

[43] Siebuhr AS, Van Der Heijde D, Bay-Jensen AC, Karsdal MA, Landewé R, Van Tubergen A (2018). Is radiographic progression in radiographic axial spondyloarthritis related to matrix metalloproteinase degradation of extracellular matrix?. RMD Open.

[44] Groen SS, Sinkeviciute D, Bay-Jensen AC, Thudium CS, Karsdal MA, Thomsen SF (2021). Exploring IL-17 in spondyloarthritis for development of novel treatments and biomarkers. Autoimmun Rev.

[45] Ademowo OS, Hernandez B, Collins E, Rooney C, Fearon U, van Kuijk AW (2016). Discovery and confirmation of a protein biomarker panel with potential to predict response to biological therapy in psoriatic arthritis. Ann Rheum Dis.

[46] Zhao B (2017). TNF and Bone Remodeling. Curr Osteoporos Rep.

[47] Wythe S, Nicolaidou V, Horwood N (2014). Cells of the immune system orchestrate changes in bone cell function. Calcif Tissue Int.

[48] Thudium CS, Nielsen SH, Sardar S, Mobasheri A, Evert Van Spil W, Lories R (2020). Bone phenotypes in rheumatology-there is more to bone than just bone. BMC Misculoskeletet Disord.

[49] Aouad K, Ziade N, Baraliakos X (2020). Structural progression in axial spondyloarthritis. Jt Bone Spine.

[50] Vossenaar ER, Radstake TRD, Van Der Heijden A, Van Mansum MAM, Dieteren C, De Rooij DJ (2004). Expression and activity of citrullinating peptidylarginine deiminase enzymes in monocytes and macrophages. Ann Rheum Dis.

